# Catatonia: demographic, clinical and laboratory associations

**DOI:** 10.1017/S0033291721004402

**Published:** 2023-04

**Authors:** Jonathan P. Rogers, Thomas A. Pollak, Nazifa Begum, Anna Griffin, Ben Carter, Megan Pritchard, Matthew Broadbent, Anna Kolliakou, Jessie Ke, Robert Stewart, Rashmi Patel, Adrian Bomford, Ali Amad, Michael S. Zandi, Glyn Lewis, Timothy R. Nicholson, Anthony S. David

**Affiliations:** 1 Division of Psychiatry, University College London, London, UK; 2 South London and Maudsley NHS Foundation Trust, London, UK; 3 Department of Psychosis Studies, King's College London, London, UK; 4 GKT School of Medical Education, King's College London, London, UK; 5 Department of Biostatistics and Health Informatics, King's College London, London, UK; 6 Department of Psychological Medicine, King's College London, London, UK; 7 Institute of Liver Studies, King's College Hospital NHS Foundation Trust, London, UK; 8 Department of Neuroimaging, King's College London, London, UK; 9 Univ. Lille, Inserm, CHU Lille, U1172 – LilNCog – Lille Neuroscience & Cognition, F-59000 Lille, France; 10 UCL Queen Square Institute of Neurology, University College London, London, UK; 11 University College London Hospitals NHS Foundation Trust, London, UK; 12 Institute of Mental Health, University College London, London, UK

**Keywords:** Catatonia, epidemiology, inflammation, NMDA receptor, mortality, admission, incidence

## Abstract

**Background:**

Catatonia, a severe neuropsychiatric syndrome, has few studies of sufficient scale to clarify its epidemiology or pathophysiology. We aimed to characterise demographic associations, peripheral inflammatory markers and outcome of catatonia.

**Methods:**

Electronic healthcare records were searched for validated clinical diagnoses of catatonia. In a case–control study, demographics and inflammatory markers were compared in psychiatric inpatients with and without catatonia. In a cohort study, the two groups were compared in terms of their duration of admission and mortality.

**Results:**

We identified 1456 patients with catatonia (of whom 25.1% had two or more episodes) and 24 956 psychiatric inpatients without catatonia. Incidence was 10.6 episodes of catatonia per 100 000 person-years. Patients with and without catatonia were similar in sex, younger and more likely to be of Black ethnicity. Serum iron was reduced in patients with catatonia [11.6 *v.* 14.2 μmol/L, odds ratio (OR) 0.65 (95% confidence interval (CI) 0.45–0.95), *p* = 0.03] and creatine kinase was raised [2545 *v.* 459 IU/L, OR 1.53 (95% CI 1.29–1.81), *p* < 0.001], but there was no difference in C-reactive protein or white cell count. *N*-Methyl-d-aspartate receptor antibodies were significantly associated with catatonia, but there were small numbers of positive results. Duration of hospitalisation was greater in the catatonia group (median: 43 *v.* 25 days), but there was no difference in mortality after adjustment.

**Conclusions:**

In the largest clinical study of catatonia, we found catatonia occurred in approximately 1 per 10 000 person-years. Evidence for a proinflammatory state was mixed. Catatonia was associated with prolonged inpatient admission but not with increased mortality.

## Introduction

Catatonia is a neuropsychiatric syndrome characterised by disturbance of volition, speech and movement with increased autonomic nervous system activity (Walther, Stegmayer, Wilson, & Heckers, 2019). It has been described as a feature of a range of other psychiatric diagnoses such as depression, mania, schizophrenia and autism (Rasmussen, Mazurek, & Rosebush, 2016; Taylor & Fink, 2003; Wing & Shah, 2000), as well as in various neurological and medical conditions such as autoimmune disorders, metabolic disturbances or viral infections (Oldham, 2018). However, despite 150 years elapsing since its first description, catatonia has been poorly studied with research findings generally based on small case series and surveys in highly heterogeneous clinical samples. In a recent meta-analysis, most studies of catatonia prevalence were small with a mean sample size of 125 (Solmi et al., 2018).

The population prevalence of catatonia has only been estimated indirectly (Taylor & Fink, 2003) and there has been a suggestion in the literature that catatonia has been observed to ‘virtually disappear’ (Mahendra, 1981). The longitudinal course of catatonia has been poorly characterised and it is not clear to what extent catatonia represents a temporary state as opposed to an underlying predisposition that manifests with periodic relapses (Walther & Strik, 2016). Gjessing described a periodic catatonia in the mid-20th century, but the most comprehensive study of catatonia relapse to date has been a case series of only 30 patients, finding that the number of episodes varied between 2 and 12 (Lin, Hung, Tsai, & Huang, 2016). One recent study found a considerably raised mortality in schizophrenia with catatonic stupor compared to other patients with schizophrenia, but this has not been generalised to catatonia as a whole (Funayama, Takata, Koreki, Ogino, & Mimura, 2018).

Previous smaller studies have suggested that rates of catatonia vary across different countries (World Health Organization, 1973) (although this was not confirmed by a recent meta-analysis) (Solmi et al., 2018) and between ethnic groups within the same country (Chandrasena, 1986). Several studies have been suggestive of higher rates among patients of Black ethnicity, but these have been limited to specific patient groups (Hutchinson, Takei, Sham, Harvey, & Murray, 1999; Lee, Schwartz, & Hallmayer, 2000), have lacked a control group (Dealberto, 2008) or have not been statistically significant (Mustafa, Bassim, Abdel Meguid, Sultan, & Al Dardiry, 2012); all have been comparatively small.

Moreover, novel research is posing new questions about the condition. For example, catatonia has been reported in encephalitis due to infections of the central nervous system (CNS) and antibodies to certain neuronal antigens (Rogers, Pollak, Blackman, & David, 2019; Samra, Rogers, Mahdi-Rogers, & Stanton, 2020). Notably, up to 88% of patients with *N*-methyl-d-aspartate (NMDA) receptor encephalitis exhibit catatonia at some point in their illness (Dalmau et al., 2008), although it is unclear whether NMDA receptor autoantibodies are present at higher rates in patients with catatonia generally. Movement disorders in NMDA receptor encephalitis are an early feature and tend to be prolonged, but it is possible that some catatonic features may be observed without the syndrome of catatonia being identified (Varley et al., 2019).

Several small studies have investigated serum iron, which initially appeared to be reduced in patients with catatonia (Carrol & Goforth, 1995; Lee, 1998), but small case–control studies have been equivocal (Haouzir et al., 2009; Lakshmana, Khanna, & Christopher, 2009; Peralta et al., 1999). Low serum iron has also been found to be predictive of neuroleptic malignant syndrome and fever after antipsychotic administration in patients with catatonia (Carrol & Goforth, 1995; Conca et al., 2003; Lee, 1998). Iron is a negative acute phase marker that is present at lower levels in acute inflammatory states and numerous autoimmune disorders have been reported with catatonia, so we have previously proposed that catatonia may also be associated with central and peripheral inflammation (Rogers et al., 2019).

This study aimed to examine the occurrence, correlates and outcomes of catatonia. Our first objective was to establish the incidence of catatonia and its relapse rate. The second objective was to investigate evidence for peripheral inflammation by comparing inpatients with catatonia to psychiatric inpatients without catatonia in terms of C-reactive protein (CRP), white cell count, creatine kinase (CK) and NMDA receptor autoantibodies. The third objective was to conduct a longitudinal comparison of patients with catatonia to psychiatric patients without catatonia in terms of their duration of hospitalisation and mortality.

## Method

### Setting

The study used the Clinical Records Interactive Search (CRIS) system, which is a large repository of anonymised electronic healthcare records from patients receiving care from the South London and Maudsley NHS Foundation Trust, UK. This Trust is the largest unit provider of secondary mental health services in the UK, serving four London boroughs with a combined 2016 population of 1 317 000, as well as providing some specialist services to the UK population nationally. Patients are seen in settings as diverse as community teams, outpatient departments, psychiatric wards and allied general hospitals. Unified electronic records were introduced between 2005 and 2006, importing previous electronic records dating back to 1999. CRIS was developed in 2008 and incorporates these previous records as well as adding current records up to the present day (Stewart et al., 2009). It currently contains records for over 500 000 individuals. The initial data extraction did not specify a time period in order to obtain the most expansive chronological data.

We used existing electronic healthcare records to identify patients with catatonia. Data were initially extracted on 17/12/2018 with subsequent data extraction occurring on 24/01/2019, 04/02/2019, 17/12/2019 and 3/09/2021. The authors assert that all procedures contributing to this work comply with the ethical standards of the relevant national and institutional committees on human experimentation and with the Helsinki Declaration of 1975, as revised in 2008. The CRIS system has approval from the Oxfordshire C Research Ethics Committee (ref: 18/SC/0372) and this study was approved by the CRIS Oversight Committee (ref: 17-102).

### Identifying patients with catatonia

To identify catatonia, we first applied a bespoke natural language processing algorithm for mentions of catatonic symptoms/syndrome, which had been developed against manually extracted gold standard annotations to a performance level of 0.86 precision (positive predictive value) and 0.87 recall (sensitivity) (Jackson et al., 2017). One of the three investigators (JPR, NB and AG) examined each positive record retrieved by the algorithm to ensure that: a diagnosis of catatonia was made by a clinician, there was a date given for the diagnosis and there was clear evidence in the case record of at least two features of catatonia as defined by the Bush–Francis Catatonia Screening Instrument (BFCSI), a tool that has a high degree of interrater reliability, construct validity, sensitivity and specificity in the identification of catatonia (Bush, Fink, Petrides, Dowling, & Francis, 1996a, 1996b; Subramaniyam, Muliyala, Suchandra, & Reddi, 2020). Where a patient had multiple episodes of catatonia, only one episode was required to list the catatonic features present.

To assess interrater reliability, 30 of the first patients' case notes were examined by more than one rater (10 by all three raters, 10 by JPR and NB and 10 by JPR and AG). Cohen's kappa coefficient for caseness on the BFCSI was 0.68, which is considered ‘substantial’ agreement (Landis & Koch, 1977). In order to assist with comparability with other studies, we also calculated the number of individuals who met DSM-5 criteria for catatonia (American Psychiatric Association, 2013).

The derivation of demographic, clinical and laboratory characteristics is described in online Supplementary eTable 1.

### Descriptive analysis

To maximise generalisability in terms of treatment setting, disease spectrum and time, all catatonia patients meeting the inclusion criteria above were included in the descriptive analyses. Index year and number of cases were assessed with Pearson's correlation. Further descriptive analyses divided annual frequency by the size of the catchment population, as estimated by the UK Office for National Statistics (Office for National Statistics, 2011). Descriptive statistics were used to investigate relapse and treatment settings.

### Case–control study

Our comparison group was drawn from the structured fields of electronic healthcare records and was composed of all individuals admitted to psychiatric wards within the same mental health trust between 2007 and 2016, covering patients with a variety of diagnoses and ages, including services treating children, adults and older people. To ensure comparability of the two groups, patients with catatonia were included only if they were inpatients on psychiatric wards admitted between these dates.

Diagnoses (other than identification of catatonia) were made according to the 10th revision of the International Statistical Classification of Diseases and Related Health Problems (ICD-10) and we reported primary diagnoses (World Health Organization, 1992). We grouped these as organic disorders (ICD-10 codes F00–F09 and non-F codes); neurodevelopmental disorders (F70–89, F90 and F95); schizophrenia and related disorders (F20–F29); mood disorders (F30–F39); neurotic disorders (F40–59); personality and behavioural disorders (F50–69, F91–F94, F98) and substance use disorders (F10–F19).

Analysis of laboratory markers was conducted in Viapath Laboratory at King's College Hospital, apart from neuronal autoantibody analyses, which were conducted in the Oxford NHS Diagnostic Neuroimmunology Service. Serum NMDA receptor antibody level results (using the Oxford live cell-based assay prior to July 2015 and the Euroimmun fixed cell-based assay thereafter) (Oxford Diagnostic Immunology Service, 2015) were officially reported by the laboratory as negative, weakly positive or positive. Due to the evidence that weakly positive peripheral antibodies can be associated with autoimmune encephalitis and high titres in the CSF (Cai et al., 2018; Qin et al., 2017), we grouped the weakly positive with the positive results to create two categories: negative and positive. Antibodies against the voltage-gated potassium channel, as measured by radioimmunoassay were reported, as data were collected prior to the reporting of antibodies to the LGi1 and CASPR2 antigens by the laboratory.

Age, sex, ethnicity, diagnostic group and laboratory markers were compared between patients with and without catatonia using logistic regression. Odds ratios (ORs) for laboratory results were calculated unadjusted and adjusted for age, sex and Black ethnicity, as these demographic factors are known to affect the results of numerous laboratory tests. Where a high degree of positive skew was present in the laboratory results, natural logarithmic transformations were used; where zero values were present, a log*_n_*(*x* + 1) transformation was used. Where the ORs for laboratory results had very narrow confidence intervals (CIs), the results were divided by their standard deviations prior to transformation.

Due to missing data in the laboratory results, the number of cases for each individual result is reported. Associations with missing data were analysed. However, multiple imputation was not conducted because computational problems can develop in data with high proportions of missing data, resulting in misleading estimates (Sterne et al., 2009).

### Cohort study

As in the case–control study, the comparison group was composed of all individuals admitted to psychiatric wards between 2007 and 2016. Patients with catatonia were included only if they were inpatients on psychiatric wards admitted between these dates. When analysing the duration of admission, patients with catatonia were included only where catatonia occurred within 7 days of admission, to avoid a bias where catatonia becomes more likely due to patients spending longer in hospital. When analysing mortality, patients with catatonia were included only where catatonia was recorded within 3 days of its onset, to avoid a survival bias in which only surviving patients would have the opportunity to have catatonia retrospectively recorded in their notes. Data were ascertained in the same way for patients with and without catatonia. Mortality data were obtained from linked national records. The time from index date to outcome (hospital discharge or death) was analysed using a Cox proportional baseline hazard model survival analysis, adjusting for age, sex, ethnicity and index year. After interaction with peer reviewers, an additional analysis adding diagnostic group as a covariate to the model was conducted. The proportionality assumption was checked using visual inspection of the Kaplan–Meier plot.

### Statistical analysis

Statistical analysis was conducted using Stata MP (version 15) with a threshold for statistical significance set to *p* < 0.05. The manuscript was written according to STROBE recommendations (checklist is given in online Supplementary eTable 2) (von Elm et al., 2007).

## Results

### Occurrence

**Fig. 1.**
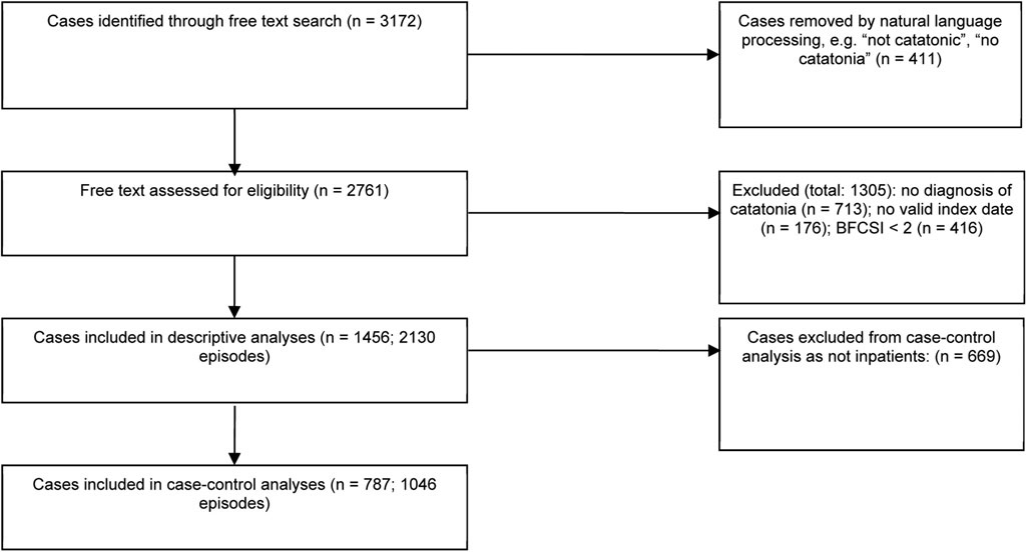
Identification and screening of cases.

**Fig. 2.**
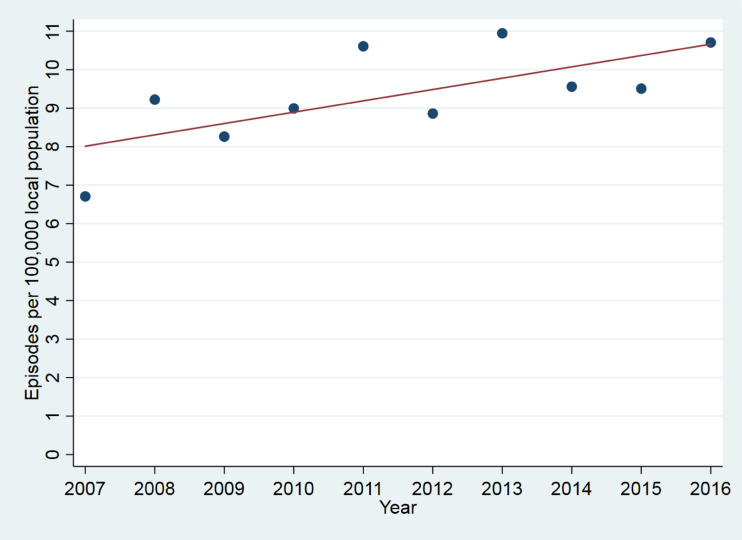
Catatonic episode per 100 000 local population by year.

The sample consisted of 2130 episodes of catatonia in 1456 subjects, as shown in Fig. 1. Overall, in the 10 year period from 2007 to 2016, among patients who were resident in the healthcare provider's catchment area, there were 1316 episodes of catatonia (852 unique patients) for the provider's catchment population of 1 242 055, giving an incidence of 10.6 (95% CI 10.0–11.1) episodes per 100 000 person-years. Where the more stringent DSM-5 criteria were used, there were 901 episodes of catatonia in 586 individuals. Among adults, there were 1214 episodes of catatonia in a mean population of 968 064, giving an incidence of 12.5 (95% CI 11.8–13.3) episodes per 100 000 person-years. Among children, there were 102 episodes of catatonia in a mean population of 273 990, giving an incidence of 3.7 (95% CI 3.0–4.5) episodes per 100 000 person-years. Examining only those episodes that were contemporaneously reported between 2007 and 2016, there was a positive correlation between index year and number of episodes (*r* = 0.70, *p* = 0.02), as shown in Fig. 2. This remained after adjusting for the mean age of the population each year (*r* = 0.71, *p* = 0.03).

Number of episodes per patient ranged between 1 and 27 (mean 1.5, s.d. 1.2, median 1, IQR 1–2) over a mean follow-up time of 7.0 years (s.d. 5.1 years). After the first episode, subsequent episodes occurred at a rate of 0.035 episodes (s.d. 0.086) per year with 25.1% experiencing at least two episodes within the follow-up period. However, after five episodes, further episodes occurred in 55.9%.

The age range for the patients at the time of first recorded diagnosis of catatonia was between 5 and 91 years (mean 35.4, median 32, s.d. 16.2, IQR 23–45 years). In terms of treatment setting at the time of diagnosis of catatonia, 1046 episodes (49.1%) were diagnosed when the individual was an inpatient in a psychiatric ward, 462 (21.7%) were in a community mental health team, 217 (10.2%) were in a general hospital, 54 (2.5%) were in a crisis resolution and home treatment team, 28 (1.3%) were in a health-based place of safety and in 323 (15.2%) the treatment setting was not specified. A total of 1022 (48.0%) were detained under the Mental Health Act for compulsory treatment within 2 weeks of the index date. The mean number of features of the BFCSI present was 3.6 (s.d. 1.7). Patients with adult and paediatric first presentation of catatonia are compared in online Supplementary eTable 3.

### Case–control study

The comparison group was drawn from all inpatients admitted to the Trust between 2007 and 2016 and represented 24 956 patients with 37 456 inpatient episodes. Demographic comparisons are made in Table 1. Patients with catatonia were similar to the control group in sex ratio but younger and more likely to be from an ethnic minority background. There were significant differences in the underlying diagnoses of the two groups.

**Table 1.** Characteristics of groups: OR for catatonia according to age, sex, ethnicity and diagnosis

	Inpatients with catatonia (*n* = 787)	Control patients (*n* = 24 956)	Unadjusted analysis		Adjusted analysis^a^	
			OR (95% CI)	*p*	aOR (95% CI)	*p*
Total number of episodes	1046	37 456	–	–	–	–
Age in years at first episode, mean (s.d.)^b^	37.0 (15.8)	40.0 (17.1)	**0.91 (0.87–0.95)**	**<0.001**	–	
Age in years at first episode, median (range, IQR)	34 (9**–**91; 24**–**46)	37 (5**–**100; 27**–**49)	**–**	**–**	–	–
Age <18 at index episode, *n* (%)	41 (5.2)	1835 (7.4)	**0.69 (0.50–0.95)**	**0.02**	–	–
Sex, *n* (%)						
Female	362 (46.0%)	11 457 (45.9%)	1 (reference)	–	1 (reference)	–
Male	425 (54.0%)	13 495 (54.1%)	0.997 (0.864**–**1.15)	0.96	–	–
Not stated	0 (0.0%)	4 (0.02%)	–	–	–	–
Ethnicity (%)						
White	233 (29.6%)	15 340 (61.5%)	1 (reference)	–	1 (reference)	–
Asian/Asian British	54 (6.9%)	1291 (5.2%)	**2.75 (2.04–3.72)**	**<0.001**	**2.68 (1.98–3.63)**	**<0.001**
Black/African/Caribbean/Black British	421 (53.5%)	6115 (24.5%)	**4.53 (3.85–5.33)**	**<0.001**	**4.45 (3.78–5.24)**	**<0.001**
Mixed/multiple ethnic groups	29 (3.7%)	609 (2.4%)	**3.14 (2.11–4.65)**	**<0.001**	**2.99 (2.01–4.44)**	**<0.001**
Other ethnic groups	47 (6.0%)	1237 (5.0%)	**2.50 (1.82–3.44)**	**<0.001**	**2.43 (1.77–3.35)**	**<0.001**
Not stated	3 (0.4%)	364 (1.5%)	–	–	–	–
Diagnostic group at first episode (%)						
Organic disorders	21 (2.7%)	1332 (5.3%)	**0.20 (0.13–0.32)**	**<0.001**	**0.32 (0.20–0.51)**	**<0.001**
Neurodevelopmental disorders	16 (2.0%)	546 (2.2%)	**0.24 (0.11–0.52)**	**<0.001**	**0.29 (0.13–0.62)**	**0.001**
Schizophrenia and related disorders	456 (57.9%)	5866 (23.5%)	1 (reference)	–	1 (reference)	–
Mood disorders	154 (19.6%)	5342 (21.4%)	**0.37 (0.31–0.45)**	**<0.001**	**0.47 (0.39–0.57)**	**<0.001**
Neurotic disorders	32 (4.1%)	2433 (9.7%)	**0.17 (0.12–0.24)**	**<0.001**	**0.22 (0.15–0.32)**	**<0.001**
Personality and behavioural disorders	18 (2.3%)	1673 (6.7%)	**0.14 (0.09–0.22)**	**<0.001**	**0.18 (0.11–0.29)**	**<0.001**
Substance use disorders	15 (1.9%)	4361 (17.5%)	**0.04 (0.03–0.07)**	**<0.001**	**0.06 (0.03–0.10)**	**<0.001**
Not stated	75 (9.5%)	3403 (13.6%)	–	–	–	–

a Ethnicity adjusted for age and sex. Diagnostic group adjusted for age, sex and Black ethnicity.

b ORs calculated using age in decades.

The main laboratory test results are compared in Table 2 with additional exploratory outcomes presented in online Supplementary eTable 4. As an additional exploratory analysis, we investigated whether serum iron and creatinine kinase were altered at baseline, or whether there was a change that corresponded to the onset of catatonia. We included all patients with catatonia who had laboratory results both for a catatonic episode and that was at least 1 month from any catatonic episode. Paired *t* tests were then used to compare the result from when the patient had catatonia with the average of the non-catatonic results. No statistically significant differences were detected between CK or iron at baseline compared to during an episode of catatonia, but numbers were small (see online Supplementary eTable 5). Comparing selected laboratory test results between patients in the catatonia group who did and did not have low serum iron revealed no significant differences after adjustment (see online Supplementary eTable 6). We also explored whether the high CK within the catatonia group was related to muscular rigidity or to rhabdomyolysis due to immobility by testing the associations between CK and each of rigidity and immobility. We found no significant relationship between CK and either of these clinical features, either in a simple measure of association or in a multivariable analysis, as shown in online Supplementary eTable 7. When receiver operating characteristic analysis was conducted for CK and catatonia diagnosis, the area under the curve was 0.64, as shown in online Supplementary eFig. 1.

**Table 2.** Laboratory results for patients with catatonia and the comparison group

Test	Patients with catatonia (*n* = 787)		Control patients (*n* = 24 955)		Unadjusted analysis		Adjusted analysis^a^	
	*n*	Mean (±s.d.)	*n*	Mean (±s.d.)	OR (95% CI)	*p*	aOR (95% CI)	*p*
Iron (μmol/L)^b^	46	11.6 (5.2)	1655	14.2 (7.9)	**0.65 (0.45–0.95)**	**0**.**03**	**0.65 (0.44–0.97)**	**0**.**04**
CK (IU/L)^c^	74	5.87 (1.42) *2545*	1881	5.20 (1.09) *459*	**1.53 (1.29–1.81)**	**<0**.**001**	**1.52 (1.27–1.83)**	**<0**.**001**
White cell count (10^9^/L)^b^	195	7.15 (2.65)	8719	7.21 (2.68)	0.98 (0.84**–**1.13)	0.76	1.05 (0.91**–**1.21)	0.68
CRP (mg/L)^c^	147	1.49 (1.07) *9.1*	5253	1.37 (0.98) *7.9*	1.13 (0.97**–**1.32)	0.13	1.14 (0.98**–**1.34)	0.10
NMDA receptor antibodies	54	Positive in 3	481	Positive in 5	**5.6 (1.3–24.1)**	**0**.**02**	**6.2 (1.4–27.3)**	**0**.**02**

a Adjusted for age, sex and ethnicity.

b Due to very small CIs, these ORs have been calculated by dividing the laboratory result by its standard deviation.

c Due to positive skew, these results underwent a natural logarithm transformation. Log*_n_* results are in normal text with original results in italics (analyses performed using log*_n_* results).

In terms of missing data, three valid laboratory values were present for 9.1% of the inpatient episodes with catatonia and 6.3% of the inpatient control episodes. When the missing data were analysed, there were significant associations with catatonic group membership, age, sex and Black ethnicity, but the absolute differences were very small. Associations are shown in online Supplementary eTable 8.

### Cohort study

When we compared the 556 episodes of catatonia (473 patients) recognised within 7 days of admission with the control group using survival analysis with hospital discharge as the outcome, we found that the baseline proportional hazards assumption was reasonable (see Kaplan–Meier plots in online Supplementary eFigs 2 and 3). The median duration of inpatient stay was 43 days (95% CI 40–49 days) among patients with catatonia, compared to 25 days (95% CI 25–26 days) in the comparison group. The unadjusted Cox proportional hazard ratio (HR) for hospital discharge was 0.77 (95% CI 0.71–0.84, *p* < 0.001); after adjusting for age, sex, Black ethnicity and year of admission, it was 0.78 (95% CI 0.72–0.85, *p* < 0.001). After the addition of diagnostic group as a covariate to the model, the HR was 0.83 (95% CI 0.76–0.90, *p* < 0.001). When the analysis was restricted to those subjects with a first episode in adulthood, the results were similar [unadjusted HR 0.77 (95% CI 0.70–0.83), *p* < 0.001; adjusted HR 0.76 (95% CI 0.69–0.83), *p* < 0.001].

When we compared the 646 patients with catatonia recorded within 3 days of its occurrence with the control group with mortality as the outcome, we found that the baseline proportional hazards assumption was reasonable (see Kaplan–Meier plot in online Supplementary eFig. 4). 3535 deaths (58 in the catatonia group) occurred during a mean follow-up time of 7.0 years (s.d. 3.2). While there was a lower mortality among patients with catatonia in the unadjusted analysis [HR 0.66 (95% CI 0.51–0.85), *p* = 0.001], after adjustment for age, sex, Black ethnicity and year of admission, there was no evidence for a difference between patients with and without catatonia [adjusted HR = 0.93 (95% CI 0.72–1.21), *p* = 0.60]. After the addition of diagnostic group as a covariate to the model, the HR was 1.12 (95% CI 0.86–1.45, *p* = 0.42). When the analysis was restricted to those subjects with a first episode in adulthood, the results were similar [unadjusted HR 0.64 (95% CI 0.49–0.83), *p* = 0.001; adjusted HR 0.92 (95% CI 0.71–1.20), *p* = 0.54].

## Discussion

This study used data from electronic patient records and is, to our knowledge, the largest clinical study on catatonia published to date (Solmi et al., 2018). Our results show that patients diagnosed with catatonia had a similar sex ratio to the general population of psychiatric inpatients, but those with catatonia were slightly younger. There was a considerable difference in ethnicity between the two groups with Black patients being substantially overrepresented among those with recorded catatonia. It has previously been proposed that this disparity is due to different interpretation of symptoms by clinicians of the dominant culture (Hutchinson et al., 1999). Other possible explanations include cultural differences in illness expression and genetic factors. It has been reported that schizophrenia is more common among migrant populations (Saha, Chant, Welham, & McGrath, 2005), but we found that the overrepresentation of schizophrenia in our catatonia population did not fully explain the ethnicity differential. Non-European origin has been shown to be a risk factor for tardive dyskinesia, another movement disorder commonly seen in psychiatric practice (Tenback, van Harten, & van Os, 2009).

Mahendra famously hypothesised, based on clinical experience, that catatonia was becoming less common (Mahendra, 1981). In this study, we found greater annual numbers of patients with catatonia between 2007 and 2016. Apart from increased recognition, one possible reason for an increase in catatonia diagnosis would be the use of certain novel psychoactive drugs (such as synthetic cannabinoids, synthetic cathinones and phenethylamines) (Mdege, Meader, Lloyd, Parrott, & McCambridge, 2017), some of which have been linked to catatonia (Khan, Pace, Truong, Gordon, & Moukaddam, 2016; Richman et al., 2018), and this will be the subject of further investigation with this dataset. Our overall incidence of catatonia (10.6 episodes per 100 000 person-years) is somewhat lower than a previous US estimate of 33.0 per 100 000 person-years that estimated rates of catatonia indirectly using proportions reported in other diagnoses (Taylor & Fink, 2003). Our figures likely represent an underestimate of the true incidence of catatonia, given that most cases of catatonia are not recognised by clinicians and catatonic signs are poorly identified (Takács, Ungvari, Antosik-Wójcińska, & Gazdag, 2021; van der Heijden et al., 2005). This may be particularly the case in a general hospital, where catatonia has been found to be common among critically ill patients (Grover, Ghosh, & Ghormode, 2014; Wilson et al., 2017); it is possible that such patients did not come to the attention of a psychiatric team.

The diagnostic heterogeneity of catatonia in our study differed somewhat from other studies in that schizophrenia and related disorders, rather than mood disorders, were most common (Taylor & Fink, 2003). It is likely that our data represent overdiagnosis of schizophrenia, as a relic of the Kraepelinian concept of catatonia as existing purely as a subtype of schizophrenia (Shorter & Fink, 2018). According to one survey of psychiatrists in Hungary, most clinicians still view catatonia as residing within the framework of schizophrenia (Takács et al., 2021). In addition, the diagnostic coding used is still ICD-10, which only formally recognises catatonia in the context of F20.2 – Catatonic schizophrenia and F06.1 – Organic catatonic disorder. Our data show that, although approximately half of cases of catatonia are recognised on psychiatric wards, appreciable rates of diagnosis occur in treatment settings such as in community teams and general hospitals. However, our data are limited to patients presenting to psychiatric services and it is likely that many cases present in general hospitals and are not assessed by a psychiatrist. We should emphasise that we report the treatment setting and whether patients were detained at the point at which catatonia was recognised, so it is possible that many patients were admitted to psychiatric hospitals shortly after catatonia recognition.

Our data on catatonia relapse show that for three quarters of patients with a first reported catatonic episode, during an average follow-up period of 7.0 years, there were no further episodes. However, there was evidence to suggest that in patients with multiple episodes, the probability of relapse is much higher, providing some evidence for Gjessing's description of periodic catatonia (Gjessing, 1932). Relapse was more common in those with an underlying psychotic disorder, although this may reflect an understanding that individuals with relapsing catatonia must necessarily be suffering from catatonic schizophrenia.

In what we believe to be the largest study of biomarkers in catatonia, we have found that iron was low relative to psychiatric controls [adjusted OR (aOR) 0.65, 95% CI 0.44–0.97] and this result remained after adjusting for demographic variables. Our initial hypothesis was that, as iron is a negative acute phase marker, this represented a peripheral inflammatory response (Rogers et al., 2019), but the lack of difference in CRP, total white cell count and erythrocyte sedimentation rate does not support this. Another possibility is that low iron is a hallmark of malnutrition, which is likely to occur in catatonia (Clinebell, Azzam, Gopalan, & Haskett, 2014) and could be a consequence of the prolonged hospitalisation. However, there was no evidence for several other markers of malnutrition – low albumin, vitamin B12, folate or creatinine (Keller, 2019; Thongprayoon, Cheungpasitporn, & Kashani, 2016) – compared to the comparison group. The relationship between iron and catatonia is therefore not clear and may relate to phenotypic heterogeneity, medication use or a more subtle immune response. It is possible that low iron creates hypokinetic symptoms by causing hypodopaminergia in the basal ganglia, as several enzymes necessary for dopamine synthesis are iron-dependent (Zucca et al., 2017); this could also explain the close association between catatonia and neuroleptic malignant syndrome (Rasmussen et al., 2016). Interestingly, low serum iron has also been demonstrated in a meta-analysis of Parkinson's disease, alongside raised iron in the substantia nigra, demonstrating that alterations in serum and CNS biometals in movement disorders are not necessarily in the same direction (Genoud, Senior, Hare, & Double, 2019). Our finding that there was no significant difference between serum iron levels during and between catatonic episodes might suggest an underlying diathesis, but it is also possible that these results were contaminated by other unrecognised episodes of catatonia.

Examining the white cell differential (online Supplementary eTable 4) suggests that the total figure masks a more complex picture. Absolute counts of neutrophils are raised (on the adjusted analysis), while lymphocytes, eosinophils and basophils are reduced. Ratios of these cell counts have more recently been used as markers of disease activity in conditions as diverse as chronic obstructive pulmonary disease, solid organ tumours, stroke and acute coronary syndromes, and their use has also been suggested for psychiatric disorders (Zulfic et al., 2020). There is evidence that the neutrophil–lymphocyte ratio (NLR), platelet–lymphocyte ratio (PLR) and monocyte–lymphocyte ratio (MLR) are higher in the manic or hypomanic phase of bipolar affective disorder than in the depressed phase (Fusar-Poli et al., 2021), while these same ratios have also been found to be higher in relapse of schizophrenia than in remission (Özdin & Böke, 2019). In catatonia, one study has found raised NLR compared to healthy controls, although there was no evidence for difference in terms of PLR and MLR. In the present study, we found NLR, MLR and PLR to be raised in catatonia relative to a psychiatric comparison group. It is possible that a relatively low lymphocyte count represents the impact of malnutrition or certain psychotropic medications (Gergely, 1999; Keller, 2019), but lymphopaenia has also been linked to autoimmunity (Schulze-Koops, 2004). The effect sizes are small, but the prospect that white cell count ratios may reflect disease activity in psychiatric disorders merits further study.

The most striking laboratory finding was the CK, where the mean result in patients with catatonia (2545 IU/L) was several times higher than that of the comparison group (459 IU/L). Three studies have previously investigated this with only one finding a significant difference, but these all used smaller samples than the present investigation (Haouzir et al., 2009; Meltzer, 1968; Northoff, Wenke, & Pflug, 1996). Raised CK may be due to muscle injury resulting from the immobility, posturing and rigidity that occur in catatonia, although the use of intramuscular injections may also have contributed. It is also possible that the group with catatonia was contaminated with patients who may have been developing neuroleptic malignant syndrome, although the result remained significant following exclusion of extreme values. Raised thyroxine in catatonia (online Supplementary eTable 4) is interesting given previous research on periodic catatonia suggesting an increased metabolic rate during episodes and a reduced rate in the intervals, which appeared to be responsive to treatment with thyroid hormones (Gjessing, 1964; Gjessing, 1975; Gunne & Gemzell, 1956), although we should note that the difference in thyroxine levels in our study was very small.

Given that almost 250 cases of catatonia have been reported co-occurring with NMDA receptor encephalitis and that catatonic features occur in up to 88% of cases of NMDA receptor encephalitis (Dalmau et al., 2008; Rogers et al., 2019), we hypothesised that NMDA receptor antibodies would be present at higher rates in the patients with catatonia. Although our data supported this hypothesis with a large OR [5.6 (95% CI 1.3–24.1)], the sample size for antibody tests was small and relied on only three positive results in the group with catatonia. It is consistent with the existing literature, where more severe catatonic features have been found in patients at ultra-high risk of psychosis who have NMDA receptor antibodies and a continuous measure of NMDA receptor immunofluorescence found higher positivity in patients with catatonia (Lin, Hung, Tsai, & Huang, 2017; Pollak et al., 2018). However, it requires replication in a larger sample.

One particularly striking finding was that patients with catatonia remained in the hospital for 134 days longer than other psychiatric inpatients, which represents an enormous degree of morbidity and a substantial economic cost. However, there was no evidence that patients with catatonia had increased mortality in multivariable analysis, in contrast to a recent Japanese study of patients with schizophrenia that found a higher mortality among those with catatonic stupor [OR 4.8 (95% CI 2.0–10.6)] (Funayama et al., 2018). The discrepancy might be explained by the restriction of the analysis in Funayama et al.'s study to patients with schizophrenia who had been hospitalised. It is possible that the mortality in our study is not elevated precisely because the patients with catatonia are more unwell than the comparison group and are treated for longer in the hospital, where their physical healthcare may be superior to the community. Moreover, some patients may have died before catatonia was formally recorded.

### Strengths and limitations

This study had several strengths, notably its large sample size, naturalistic data, psychiatric control group, rigorous standards for defining catatonia and linkage to national records to define mortality. However, using routine clinical records also brings several disadvantages, including a reliance on clinician identification of disorders and symptoms, which means that incidences are likely underestimated, perhaps especially in general hospitals, where our figures were particularly low. Moreover, it is likely that a reliance on clinician identification will preferentially exclude patients without classical features of catatonia, such as those with excited or less acute presentations. Prospective studies, which continue to take place (Espinola-Nadurille et al., 2019; Sarkar et al., 2016), are able to avoid this bias, although case numbers tend to be much smaller.

In terms of measurement, although the Bush–Francis has previously been used in paediatric populations (Grover, Chauhan, Sharma, Chakrabarti, & Avasthi, 2017), it has not been specifically validated in this group (Benarous et al., 2016).

Regarding detection of markers of autoimmunity, we were limited to serum samples, but there is evidence that titres are higher in cerebrospinal fluid, thus potentially conferring greater sensitivity (Dalmau et al., 2008).

In terms of missing data, the epidemiological estimates may also have been biased by patients who were lost to follow-up by moving out of the area; this may have preferentially affected individuals who are less unwell. The missing diagnoses may differ from the available diagnoses and this is likely, as clinical presentation may affect the probability of making or recording a diagnosis. The fact that only a minority of patients had a valid laboratory result for any given test means that selection bias is possible and exploration of the missing data did suggest small but statistically significant associations with ethnicity and membership of the catatonia group.

In terms of confounding, there was evidence of confounding by age, sex and ethnicity, but after adjusting for these factors, the major findings remained. Given the high proportion of individuals from a Black ethnic group in the catchment area for the service and our finding that Black patients were more likely to have catatonia, this may have inflated our estimates of incidence above what is nationally representative. One important unmeasured confounder in the laboratory test results is medication use, which means it is not possible to establish if differences in laboratory test results are due to intramuscular injection or the development of neuroleptic malignant syndrome.

Finally, despite the large sample size, due to the relatively low number of deaths in the catatonia group, our statistical power for detecting a difference in mortality between the two groups was limited. It is possible that some of the findings arose by chance, given that several hypotheses were tested.

## Conclusions

This study demonstrates that catatonia remains an important clinical problem with significant morbidity and may even be increasing in frequency of diagnosis in certain areas. Catatonia features in a large array of psychiatric diagnoses and is associated with a very prolonged hospital admission. In terms of biomarkers, there remains evidence for low serum iron and raised CK, but prospective controlled studies are necessary for confirmation.

## Data Availability

A data dictionary defining each variable used in the study is available in Supplementary eTable 1. Data are owned by a third party, Maudsley Biomedical Research Centre (BRC) Clinical Records Interactive Search (CRIS) tool, which provides access to anonymised data derived from South London and Maudsley NHS Foundation Trust electronic medical records. These data can only be accessed by permitted individuals from within a secure firewall (i.e. the data cannot be sent elsewhere), in the same manner as the authors. For more information please contact: cris.administrator@slam.nhs.uk.

## Supplementary material

For supplementary material accompanying this paper visit https://doi.org/10.1017/S0033291721004402. click here to view supplementary material
