# Supplementary material for: Catatonia: demographic, clinical and laboratory associations
Source: Psychol Med. 2021 Nov 2;53(6):2492–502. doi: 10.1017/S0033291721004402 (PMC10123832; doi:10.1017/S0033291721004402)

# Online Supplementary Material

Contents

[eTable 1: Variable properties 1](#_Toc83980061)

[eTable 2: STROBE checklist 2](#_Toc83980062)

[eTable 3: Comparison of demographic and clinical data for adult and paediatric patients with catatonia 6](#_Toc83980063)

[eTable 4: Additional exploratory laboratory results for patients with catatonia and without catatonia 7](#_Toc83980064)

[eTable 5: Longitudinal comparison of creatine kinase and iron in patients with catatonia 8](#_Toc83980065)

[eTable 6: Comparison of patients with catatonia with and without low serum iron 8](#_Toc83980066)

[eTable 7: Association of creatine kinase with rigidity and immobility 8](#_Toc83980067)

[eTable 8: Comparison of associations with missing and non-missing data (as measured by inpatients having 3 or more valid laboratory test results) 9](#_Toc83980068)

[eFigure 1: Receiver operating charcteristic curve for CK and diagnosis of catatonia 10](#_Toc83980069)

[eFigure 2: Unadjusted Kaplan-Meier curve for hospital discharge 11](#_Toc83980070)

[eFigure 3: Unadjusted Kaplan-Meier curve for hospital discharge restricted to first 100 days 12](#_Toc83980071)

[eFigure 4: Unadjusted Kaplan-Meier curve for mortality 13](#_Toc83980072)

## eTable 1: Variable properties

| Variable | | Structured field | NLP-derived | Free text analysed by researcher | Measurement |
| --- | --- | --- | --- | --- | --- |
| Demographic | Age | ⚫ |  |  | For patients with catatonia, this was age at index date. For comparison group, this was age on 1^st^ June of the year they were admitted as an inpatient. |
|  | Date of birth | ⚫ |  |  | Adjusted to first date of the month to preserve anonymisation |
|  | Date of death | ⚫ |  |  | Linked to NHS Spine |
|  | Ethnicity | ⚫ |  |  | Dichotomised as Black and not Black when used for adjustment in regression analyses |
|  | Sex | ⚫ |  |  |  |
|  | Index date | ⚫ |  |  | Date of admission to hospital |
| Diagnosis | Presence of catatonia |  |  | ⚫ | According to Bush-Francis Catatonia Screening Instrument |
|  | Diagnosis | ⚫ |  |  | Where an ICD-10 diagnosis had been coded prior to the index date, the most recent diagnostic code prior to the index date was used. Where there was no diagnostic code prior to the index, the earliest diagnostic code up to 6 months after the index date was used. |
| Treatment | Date of first referral accepted | ⚫ |  |  | First date on which a referral to the Trust was accepted |
|  | Admission date | ⚫ |  |  | Date of admission to hospital |
|  | Discharge date | ⚫ |  |  | Date of discharge from hospital |
|  | Detention under the Mental Health Act | ⚫ |  |  | Included any active inpatient section from the index date until 2 weeks later |
|  | Health of the Nation Outcomes Scale (HoNOS) | ⚫ |  |  | Latest before index date and earliest after index date. |
| Blood pressure | Systolic blood pressure |  | ⚫ |  | Earliest blood pressure within 2 weeks of index date |
|  | Diastolic blood pressure |  | ⚫ |  |  |
| Laboratory results | Full blood count | ⚫ |  |  | Earliest from index date to 14 days later |
|  | Urea and electrolytes | ⚫ |  |  |  |
|  | Thyroid function and autoantibodies | ⚫ |  |  |  |
|  | Iron studies | ⚫ |  |  |  |
|  | Vitamin B12 and folate | ⚫ |  |  |  |
|  | Creatine kinase | ⚫ |  |  |  |
|  | D-dimer | ⚫ |  |  |  |
|  | Autoantibody profile | ⚫ |  |  | Earliest from index date to 1 year |

## eTable 2: STROBE checklist

|  | Item No | Recommendation | Location |
| --- | --- | --- | --- |
| **Title and abstract** | 1 | (*a*) Indicate the study’s design with a commonly used term in the title or the abstract | - Abstract |
|  |  | (*b*) Provide in the abstract an informative and balanced summary of what was done and what was found | - Abstract |
| Introduction | | | |
| Background/rationale | 2 | Explain the scientific background and rationale for the investigation being reported | - Introduction |
| Objectives | 3 | State specific objectives, including any prespecified hypotheses | - Introduction |
| Methods | | | |
| Study design | 4 | Present key elements of study design early in the paper | - Method |
| Setting | 5 | Describe the setting, locations, and relevant dates, including periods of recruitment, exposure, follow-up, and data collection | - Method/Setting |
| Participants | 6 | (*a*) *Cohort study*—Give the eligibility criteria, and the sources and methods of selection of participants. Describe methods of follow-up  *Case-control study*—Give the eligibility criteria, and the sources and methods of case ascertainment and control selection. Give the rationale for the choice of cases and controls  *Cross-sectional study*—Give the eligibility criteria, and the sources and methods of selection of participants | - Method/Identifying patients with catatonia; Descriptive analyses; Cases-control study; Cohort study |
|  |  | (*b*) *Cohort study*—For matched studies, give matching criteria and number of exposed and unexposed  *Case-control study*—For matched studies, give matching criteria and the number of controls per case | - N/A |
| Variables | 7 | Clearly define all outcomes, exposures, predictors, potential confounders, and effect modifiers. Give diagnostic criteria, if applicable | - Method/Identifying patients with catatonia; Descriptive analyses; Cases-control study; Cohort study - eTable 1 |
| Data sources/ measurement | 8* | For each variable of interest, give sources of data and details of methods of assessment (measurement). Describe comparability of assessment methods if there is more than one group | - Method/Identifying patients with catatonia; Descriptive analyses; Cases-control study; Cohort study - eTable 1 |
| Bias | 9 | Describe any efforts to address potential sources of bias | - Method/Cohort study |
| Study size | 10 | Explain how the study size was arrived at | - Method/Identifying patients with catatonia - Figure 1 |
| Quantitative variables | 11 | Explain how quantitative variables were handled in the analyses. If applicable, describe which groupings were chosen and why | - Method/Identifying patients with catatonia; Descriptive analyses; Cases-control study; Cohort study |
| Statistical methods | 12 | (*a*) Describe all statistical methods, including those used to control for confounding | - Method/Identifying patients with catatonia; Descriptive analyses; Cases-control study; Cohort study; Statistical analysis |
|  |  | (*b*) Describe any methods used to examine subgroups and interactions | - N/A |
|  |  | (*c*) Explain how missing data were addressed | - Method/Case-control study |
|  |  | (*d*) *Cohort study*—If applicable, explain how loss to follow-up was addressed  *Case-control study*—If applicable, explain how matching of cases and controls was addressed  *Cross-sectional study*—If applicable, describe analytical methods taking account of sampling strategy | - Method/Cohort study |
|  |  | (*e*) Describe any sensitivity analyses | - Method/Cohort study |

| Results | | | |
| --- | --- | --- | --- |
| Participants | 13* | (a) Report numbers of individuals at each stage of study—eg numbers potentially eligible, examined for eligibility, confirmed eligible, included in the study, completing follow-up, and analysed | - Figure 1 |
|  |  | (b) Give reasons for non-participation at each stage | - Figure 1 |
|  |  | (c) Consider use of a flow diagram | - Figure 1 |
| Descriptive data | 14* | (a) Give characteristics of study participants (eg demographic, clinical, social) and information on exposures and potential confounders | - Table 1 |
|  |  | (b) Indicate number of participants with missing data for each variable of interest | - Table 1 |
|  |  | (c) *Cohort study*—Summarise follow-up time (eg, average and total amount) | - Results/Cohort study |
| Outcome data | 15* | *Cohort study*—Report numbers of outcome events or summary measures over time | - Results/Cohort study |
|  |  | *Case-control study—*Report numbers in each exposure category, or summary measures of exposure | - Results/Case-control study - Table 2 |
|  |  | *Cross-sectional study—*Report numbers of outcome events or summary measures | - N/A |
| Main results | 16 | (*a*) Give unadjusted estimates and, if applicable, confounder-adjusted estimates and their precision (eg, 95% confidence interval). Make clear which confounders were adjusted for and why they were included | - Results/Case-control study; Cohort study - Table 2 |
|  |  | (*b*) Report category boundaries when continuous variables were categorized | - N/A |
|  |  | (*c*) If relevant, consider translating estimates of relative risk into absolute risk for a meaningful time period | - N/A |
| Other analyses | 17 | Report other analyses done—eg analyses of subgroups and interactions, and sensitivity analyses | - Results/Case-control study - eTables 3-8 |
| Discussion | | | |
| Key results | 18 | Summarise key results with reference to study objectives | - Discussion |
| Limitations | 19 | Discuss limitations of the study, taking into account sources of potential bias or imprecision. Discuss both direction and magnitude of any potential bias | - Discussion/Strengths and limitations |
| Interpretation | 20 | Give a cautious overall interpretation of results considering objectives, limitations, multiplicity of analyses, results from similar studies, and other relevant evidence | - Conclusions |
| Generalisability | 21 | Discuss the generalisability (external validity) of the study results | - Discussion |
| Other information | | | |
| Funding | 22 | Give the source of funding and the role of the funders for the present study and, if applicable, for the original study on which the present article is based | - Source of funding - Method/Role of the funding source |

## eTable 3: Comparison of demographic and clinical data for adult and paediatric patients with catatonia

|  |  | Paediatric presentation (1^st^ episode at <18 years) | Adult presentation (1^st^ episode ≥18 years) | Total sample |
| --- | --- | --- | --- | --- |
|  | **Number of patients** | 119 | 1,337 | 1,456 |
|  | **Number of episodes** | 203 | 1,927 | 2,130 |
|  | **Number of episodes per patient, median (IQR)** | 1 (1 to 2) | 1 (1 to 1) | 1 (1 – 2) |
|  | **Number of episodes per patient, mean (SD)** | 1.7 (2.6) | 1.4 (1.0) | 1.5 (1.2) |
| Figures provided per patient (first episode) | **Age at first episode, mean (s.d.)** | 14.6 (2.7) | 37.3 (15.6) | 35.4 (16.2) |
|  | **Age at first episode, median (range, IQR)** | 15 (5 to 17; 14 to 17) | 34 (18 to 91; 25 to 46) | 32 (5 to 91; 23 to 45) |
|  | **Sex (*n*, %)** |  |  |  |
|  | - Male | 77 (64.7) | 726 (54.3) | 803 (55.2) |
|  | - Female | 42 (35.3) | 611 (45.7) | 653 (44.9) |
|  | **Ethnicity (*n*, %)** |  |  |  |
|  | - White | 28 (23.5) | 469 (35.1) | 497 (34.1) |
|  | - Asian / Asian British | 12 (10.1) | 81 (6.1) | 93 (6.4) |
|  | - Black / African / Caribbean / Black British | 55 (46.2) | 646 (48.3) | 701 (48.1) |
|  | - Mixed / Multiple ethnic groups | 10 (8.4) | 39 (2.9) | 49 (3.4) |
|  | - Other ethnic groups | 10 (8.4) | 77 (5.8) | 87 (6.0) |
|  | - Not stated | 4 (3.4) | 25 (1.9) | 29 (2.0) |
|  | **BFCSI score, median (IQR)** | 3 (2-5) | 3 (2-4) | 3 (2-5) |
|  | **BFCSI score, mean (SD)** | 3.9 (1.9) | 3.6 (1.7) | 3.6 (1.7) |
| Figures provided per episode | **Treatment setting (*n*, %)** |  |  |  |
|  | - Psychiatric ward | 69 (34.0) | 977 (50.7) | 1,046 (49.1) |
|  | - Community mental health team | 72 (35.5) | 390 (20.2) | 462 (21.7) |
|  | - General hospital | 11 (5.4) | 206 (10.7) | 217 (10.2) |
|  | - Crisis resolution and home treatment team | 2 (1.0) | 52 (2.7) | 54 (2.5) |
|  | - Health-based place of safety | 0 (0.0) | 28 (1.5) | 28 (1.3) |
|  | - Not specified | 49 (24.1) | 274 (14.2) | 323 (15.2) |
|  | **Detention under Mental Health Act for compulsory treatment within 2 weeks of index date (*n*, %)** |  |  |  |
|  | - Detained | 58 (28.6) | 964 (50.0) | 1,022 (48.0) |
|  | - Not detained | 145 (71.4) | 963 (50.0) | 1,108 (52.0) |

## eTable 4: Additional exploratory laboratory results for patients with catatonia and without catatonia

| Test | Patients with catatonia (n=787) | | Control patients (n=24,956) | | Unadjusted analysis | | Adjusted analysis ^a^ | |
| --- | --- | --- | --- | --- | --- | --- | --- | --- |
|  | *n* | Mean (+/- S.D.) | *n* | Mean (+/- S.D.) | OR (95% CI) | *p* | aOR (95% CI) | *p* |
| Erythrocyte sedimentation rate (mm/hr) ^b^ | 28 | 14.1 (11.9) | 1146 | 14.8 (18.4) | 0.96 (0.65 to 1.42) | 0.84 | 0.87 (0.55 to 1.37) | 0.54 |
| Full blood count |  |  |  |  |  |  |  |  |
| - Haemoglobin (g/L) ^b^ | 195 | 134 (16) | 8723 | 137 (16) | **0.82 (0.72 to 0.94)** | **0.004** | 0.93 (0.79 to 1.09) | 0.35 |
| - Mean corpuscular volume (fL) ^b^ | 195 | 88.6 (7.3) | 8723 | 91.2 (7.1) | **0.71 (0.62 to 0.81)** | **<0.001** | **0.76 (0.66 to 0.87)** | **<0.001** |
| - Neutrophil count (10^9^/L) ^b^ | 195 | 4.69 (2.38) | 8719 | 4.49 (2.15) | 1.09 (0.95 to 1.24) | 0.22 | **1.18 (1.04 to 1.34)** | **0.01** |
| - Lymphocyte count (10^9^/L) | 195 | 1.86 (0.70) | 8719 | 2.05 (1.03) | **0.70 (0.57 to 0.86)** | **0.001** | **0.67 (0.54 to 0.83)** | **<0.001** |
| - Monocyte count (10^9^/L) | 195 | 0.46 (0.19) | 8719 | 0.46 (0.19) | 0.89 (0.41 to 1.92) | 0.77 | 1.31 (0.61 to 2.81) | 0.50 |
| - Eosinophil count (10^9^/L) ^b^ | 195 | 0.12 (0.09) | 8717 | 0.17 (0.16) | **0.58 (0.46 to 0.73)** | **<0.001** | **0.61 (0.48 to 0.77)** | **<0.001** |
| - Basophil count (10^9^/L) ^b^ | 195 | 0.032 (0.020) | 8705 | 0.038 (0.024) | **0.73 (0.60 to 0.87)** | **0.001** | **0.79 (0.66 to 0.95)** | **0.012** |
| - Platelets (10^9^/L) ^b^ | 195 | 266 (78) | 8723 | 262 (87) | 1.05 (0.91 to 1.20) | 0.52 | 1.02 (0.89 to 1.18) | 0.74 |
| - Neutrophil-lymphocyte ratio | 195 | 3.0 (2.6) | 8719 | 2.5 (1.6) | **1.13 (1.07 to 1.19)** | **<0.001** | **1.16 (1.10 to 1.23)** | **<0.001** |
| - Monocyte-lymphocyte ratio | 195 | 0.27 (0.14) | 8719 | 0.25 (0.12) | **3.47 (1.45 to 8.30)** | **0.005** | **6.02 (2.54 to 14.2)** | **<0.001** |
| - Platelet-lymphocyte ratio ^b^ | 195 | 163 (83) | 8719 | 144 (72) | **1.21 (1.09 to 1.34)** | **<0.001** | **1.22 (1.10 to 1.36)** | **<0.001** |
| Thyroid function |  |  |  |  |  |  |  |  |
| - Free T_4_ (pmol/L) ^b^ | 140 | 15.9 (3.37) | 8027 | 14.8 (3.0) | **1.20 (1.08 to 1.34)** | **0.001** | **1.20 (1.06 to 1.35)** | **0.003** |
| - Thyroid stimulating hormone (mIU/L) ^c^ | 140 | 0.84 (0.54)  *2.0* | 7953 | 0.90 (0.42)  *1.8* | 0.69 (0.44 to 1.07) | 0.10 | 0.74 (0.48 to 1.15) | 0.18 |
| Haematinics |  |  |  |  |  |  |  |  |
| - Ferritin (µg/L) ^c^ | 96 | 4.41 (1.10)  *135* | 4588 | 4.33 (1.04)  *137* | 1.08 (0.89 to 1.31) | 0.44 | 1.16 (0.94 to 1.44) | 0.16 |
| - Vitamin B12 (ng/L) ^b^ | 120 | 553 (291) | 6959 | 498 (258) | **1.19 (1.03 to 1.37)** | **0.02** | 1.11 (0.95 to 1.29) | 0.20 |
| - Folate (µg/L) ^b^ | 117 | 8.8 (6.1) | 6624 | 8.8 (5.6) | 1.02 (0.85 to 1.22) | 0.86 | 1.01 (0.84 to 1.21) | 0.92 |
| Albumin (g/L) | 188 | 43.4 (4.0) | 7978 | 43.9 (3.7) | 0.97 (0.93 to 1.00) | 0.09 | 0.99 (0.95 to 1.03) | 0.48 |
| Creatinine (µmol/L) | 192 | 77.7 (46.3) | 8030 | 74.0 (39.2) | 1.00 (1.00 to 1.00) | 1.00 (1.00 to 1.00) |  |  |
| ‘Voltage-gated potassium channel’ antibodies (pM/L) ^c^ | 42 | 1.73 (1.92)  *73* | 389 | 2.05 (1.73)  *37* | 0.90 (0.74 to 1.09) | 0.27 | 0.88 (0.73 to 1.08) | 0.23 |

^a^Adjusted for age, sex and ethnicity. ^b^ Due to very small confidence intervals, these odds ratios have been calculated by dividing the laboratory result by its standard deviation. ^c^ Due to positive skew, these results underwent a natural logarithm transformation. Log_n_ results are in normal text with original results in italics (analyses performed using log_n_ results)

## eTable 5: Longitudinal comparison of creatine kinase and iron in patients with catatonia

| **Laboratory result** | ***n*** | **Result when catatonia present, mean (+/- S.D.)** | **Result when catatonia not present (+/- S.D.)** | **Mean difference (95% CI)** | ***p*** |
| --- | --- | --- | --- | --- | --- |
| Creatine kinase ^a^ | 20 | 6.3 (1.7) | 5.6 (1.1) | 0.7 (-0.1 to 1.5) | 0.08 |
| Iron | 15 | 9.5 (3.5) | 11.9 (4.6) | -2.4 (-5.4 to 0.6) | 0.11 |

^a^ Due to positive skew, creatine kinase results underwent a natural logarithm transformation.

## eTable 6: Comparison of patients with catatonia with and without low serum iron

|  | Serum iron low (*n=33)* | | Serum iron normal or high (*n=13)* | | Unadjusted analysis | | Adjusted analysis ^a^ | |
| --- | --- | --- | --- | --- | --- | --- | --- | --- |
|  | *n* | Mean (+/-SD) | *n* | Mean (+/-SD) | OR (95% CI) | *p* | OR (95% CI) | *p* |
| Haemoglobin (g/L) | 32 | 130 (16) | 12 | 139 (20) | 0.97 (0.93 to 1.01) | 0.14 | 1.00 (0.95 to 1.06) | 0.92 |
| White cell count (10^9^/L) | 32 | 7.0 (2.4) | 12 | 6.8 (2.4) | 1.0 (0.8 to 1.4) | 0.85 | 1.1 (0.7 to 1.7) | 0.63 |
| C-reactive protein (mg/L) ^b^ | 28 | 1.7 (1.2)  *11* | 9 | 1.1 (0.8)  *5* | 1.8 (0.7 to 4.3) | 0.22 | 1.3 (0.5 to 3.5) | 0.58 |
| Erythrocyte sedimentation rate (mm/hr) | 5 | 19 (11) | 4 | 14 (16) | 1.0 (0.9 to 1.2) | 0.50 | 0.6 (0.1 to 2.0) | 0.41 |
| Ferritin (µg/L) ^b^ | 32 | 4.6 (1.0)  *168* | 11 | 4.6 (1.0)  *129* | 1.0 (0.5 to 2.1) | 0.92 | 2.0 (0.6 to 6.7) | 0.25 |
| Albumin (g/L) | 33 | 43 (3) | 12 | 46 (4) | 0.75 (0.58 to 0.96) | 0.03 | 0.87 (0.65 to 1.15) | 0.32 |
| NMDA receptor antibodies | 6 | Negative in 6 | 1 | Negative in 1 | - | - | - | - |

^a^ Adjusted for age, sex and Black ethnicity. ^b^ Due to positive skew, these results underwent a natural logarithm transformation. Log_n_ results are in normal text with original results in italics (analyses performed using log_n_ results)

## eTable 7: Association of creatine kinase with rigidity and immobility

| Creatine kinase (IU/L) ^a^ | Patients with catatonia (n=787) | | Unadjusted analysis | | Adjusted analysis ^b^ | |
| --- | --- | --- | --- | --- | --- | --- |
|  | *n* | Mean (+/- S.D.) | OR (95% CI) | *p* | aOR (95% CI) | *p* |
| Rigidity   - Present - Absent | 20  54 | 5.66  5.94 | 0.86 (0.58 to 1.27) | 0.45 | 0.80 (0.53 to 1.22) | 0.30 |
| Immobility / stupor   - Present - Absent | 51  23 | 5.82  5.98 | 0.92 (0.66 to 1.30) | 0.66 | 0.95 (0.66 to 1.35) | 0.76 |

^a^ Due to positive skew, creatine kinase underwent a natural logarithm transformation. ^b^ Adjusted for age, sex and ethnicity.

## eTable 8: Comparison of associations with missing and non-missing data (as measured by inpatients having 3 or more valid laboratory test results)

|  |  | **n(%) missing** |  | **n(%) missing** | ***p*** |
| --- | --- | --- | --- | --- | --- |
| **Sex** | Male | 19,646 (94.2) | Female | 16,404 (93.0) | <0.001 |
| **Ethnicity** | Black | 10,675 (93.2) | Not Black | 25,046 (93.9%) | 0.009 |
| **Group** | Catatonia | 951 (90.9) | Comparison | 35,103 (93.7) | <0.001 |
|  |  |  |  |  |  |
|  | | **n(%) missing** | **Mean (+/-) SD for missing** | **Mean (+/-) SD for not missing** | ***p*** |
| **Age (years)** | | 36,051 (93.6) | 40.1 (16.2) | 39.1 (16.5) | 0.002 |

## eFigure 1: Receiver operating characteristic curve for CK and diagnosis of catatonia


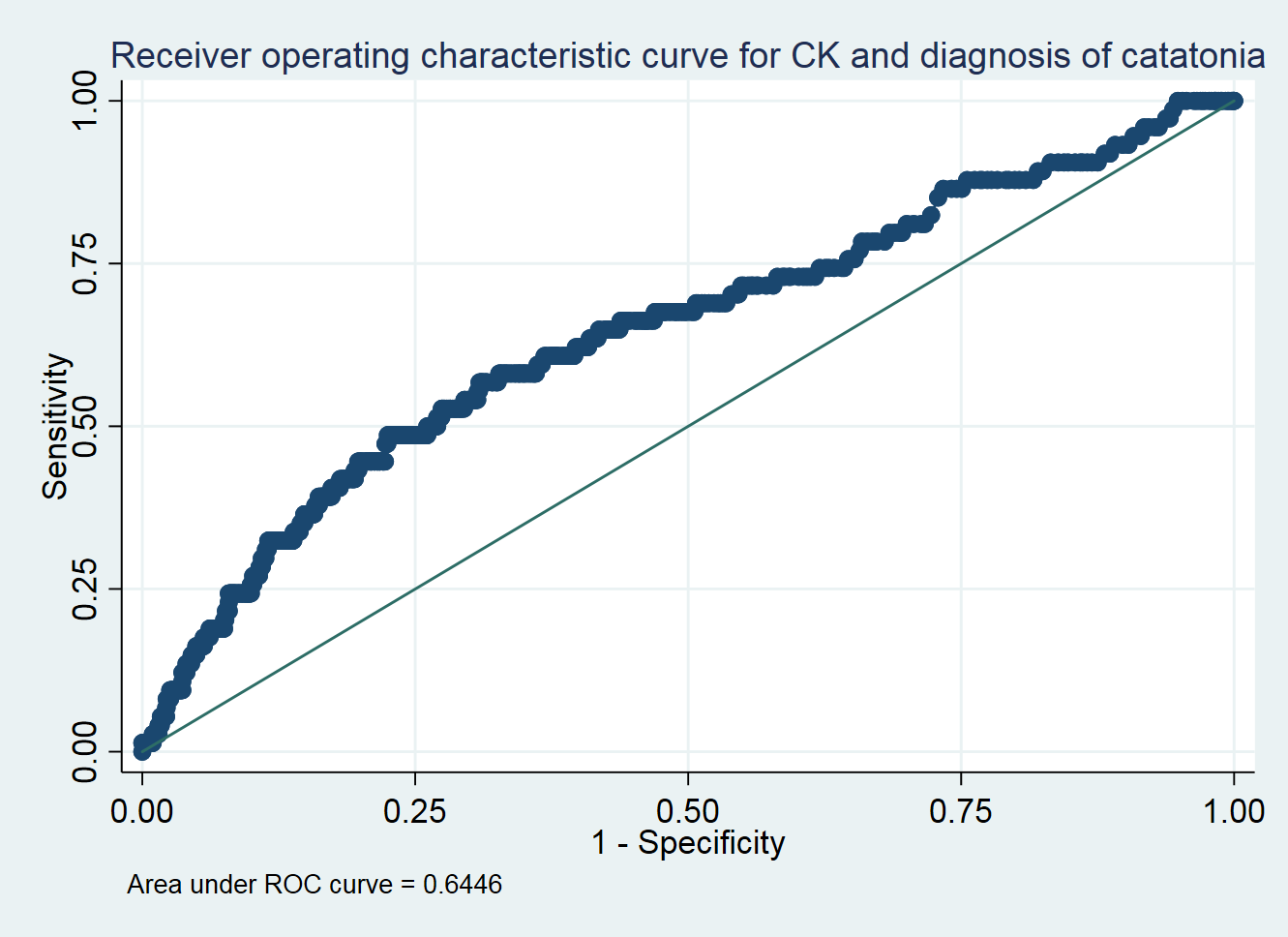


## eFigure 2: Unadjusted Kaplan-Meier curve for hospital discharge


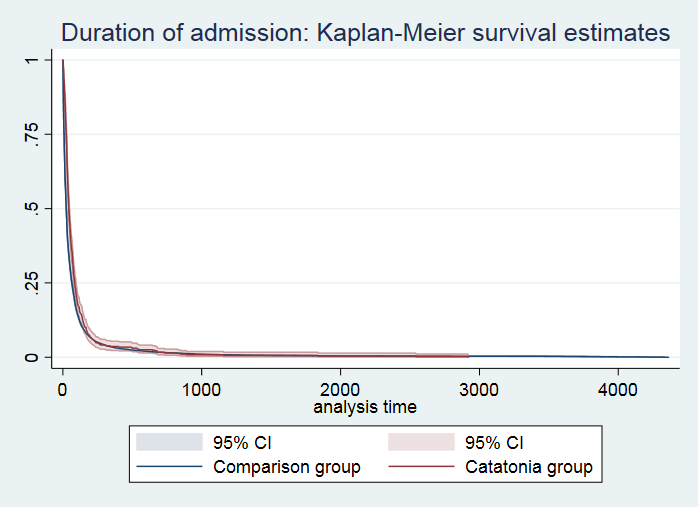


## eFigure 3: Unadjusted Kaplan-Meier curve for hospital discharge restricted to first 100 days


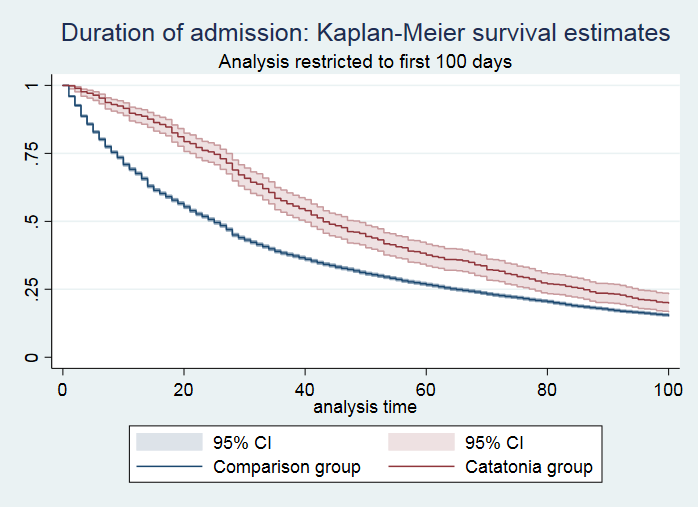


## eFigure 4: Unadjusted Kaplan-Meier curve for mortality


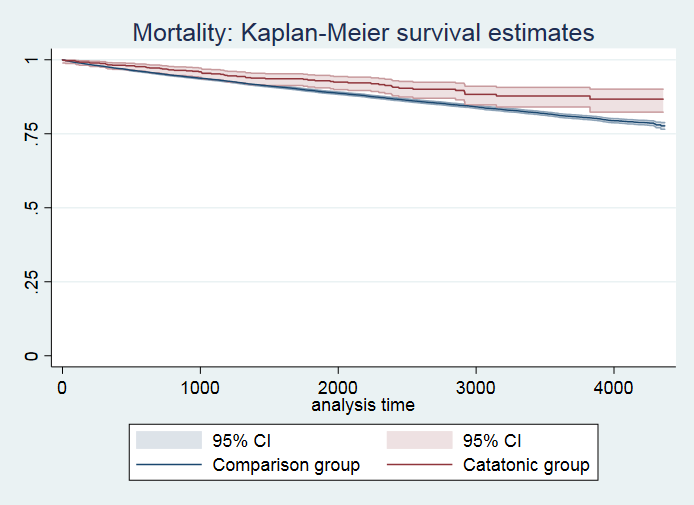

Supplement: Supplementary file 1 [file S0033291721004402sup001.docx]
